# MgSO_4_ alleviates hippocampal neuroinflammation and BBB damage to resist CMS-induced depression

**DOI:** 10.3389/fnut.2025.1470505

**Published:** 2025-03-26

**Authors:** Qiaona Wang, Yuefeng Hu, Fan Li, Liyun Hu, Yizhu Zhang, Yunfa Qiao, Chuanfeng Tang, Renlei Wang

**Affiliations:** ^1^School of Ecology and Applied Meteorology, Nanjing University of Information Science and Technology, Nanjing, China; ^2^Biology Department, Jiangsu Second Normal University, Nanjing, China; ^3^School of Food Science and Pharmaceutical Engineering, Nanjing Normal University, Nanjing, China; ^4^State Key Laboratory on Technologies for Chinese Medicine Pharmaceutical Process Control and Intelligent Manufacture, Nanjing University of Chinese Medicine, Nanjing, China

**Keywords:** CMS, MgSO_4_, hippocampal neuroinflammation, blood-brain barrier, depression

## Abstract

**Purpose:**

Magnesium sulfate (MgSO_4_) possesses the advantages of being readily accessible, cost-effective, and having low toxicity. It has potential applications as a neuroprotective agent. The mechanisms underlying the effects of Mg^2+^ treatment on depression and its neuroprotective properties remain poorly elucidated.

**Methods:**

In this study, we employed chronic mild unpredictable stress (CMS)-induced mice were orally administered with MgSO_4_ or pioglitazone. The CMS-induced depressive-like behaviors of mice were monitored. After sacrifice, the levels of Mg^2+^ and inflammatory cytokines were observed. Blood-brain barrier (BBB) permeability and the M1-to-M2 shift of microglia in mouse hippocampus were detected. The expression of proteins in IKK/NF-κB and NLRP3 inflammasome signal pathway were analyzed.

**Results:**

We found that CMS induced depressive-like behaviors as well as hypomagnesemia in mice, which were accompanied with hypersecretion of inflammatory cytokines in hippocampus of mice. These animals induced by CMS exhibited hippocampal neuroinflammation characterized by an elevated number of Iba^+^ microglia with enlarged cell bodies and increased branching structures. In CMS-induced mice, MgSO_4_ alleviated CMS-induced depressive-like behaviors and hypomagnesemia, reduced the levels of inflammatory cytokines in both serum and hippocampus, decreased the number of Iba^+^ microglia, modulated microglia polarization and repaired the BBB damage. MgSO_4_ also significantly facilitates the M1-to-M2 shift in CMS-induced mouse hippocampus and lipopolysaccharide (LPS)-induced BV2 microglia. Mechanically, we found that MgSO_4_ inhibited microglia activation and BBB damage, possibly by suppressing IKK/NF-κB and NLRP3 inflammasome signaling pathways.

**Conclusion:**

Our findings showed that MgSO_4_ supplementation played an active role in the prevention and treatment of depression.

## 1 Introduction

Depression, characterized by persistent feelings of sadness, is the most common mental health disorder with emotional disturbances. It is currently the leading cause of disability worldwide (1, 2). Antidepressants should be considered for their active role in reducing the duration and severity of current depressive episodes, as well as lowering the risk of recurrent episodes. The most commonly used antidepressants in clinical practice belong to the second-generation class, which includes serotonin-norepinephrine reuptake inhibitors (SNRIs), selective serotonin reuptake inhibitors (SSRIs), and atypical antidepressants. However, second-generation antidepressants have been found to have similar or lower efficacy compared to first-generation and non-first-line antidepressants such as tricyclic antidepressants (TCAs) and monoamine oxidase inhibitors (MAOIs). First-generation antidepressants are known to have less receptor specificity, resulting in a wider range of side effects and a higher potential for toxicity (3). In addition, antidepressants may be associated with an increased risk of suicide in children, adolescents, and young adults (4). A meta-analysis study that included 2,741 patients aged 6–18 years revealed an increased relative risk of self-harm or suicide-related events in patients treated with newer-generation antidepressants (SSRIs, venlafaxine, and mirtazapine) (4). Therefore, there is an urgent need for new treatment approaches with high efficacy and minimal side-effect profiles.

Selecting a stable and appropriate animal model of depression is crucial for basic experimental research. Many animal models of depression have been developed and improved over time, each with its own set of attributes and limitations (5–7). At the end of the 20th century, Willner (8) established a chronic mild unpredictable stress (CMS) model that reliably and effectively mimics the life stress experienced by people. This model is widely utilized to induce depressive-like phenotypes in rodents (9, 10). In neurological system, brain injury or emotional disorder is often accompanied by the activation of microglia and the development of neuroinflammation (11–13). Activated microglial cells undergo morphological transformation, characterized by an increase in the size of cell bodies and a decrease in the ramification of distal branches. These cells also secrete pro-inflammatory cytokines, contributing to self-perpetuating damage to neurons, a state known as the classically activated M1 phenotype (14, 15). However, an alternatively activated M2 phenotype can serve as neurosupport and neuroprotection (16, 17).

Mg^2+^ currently is suggested as a neuroprotective factor (18, 19). Hypomagnesemia is prevalent and accounts for approximately 14.5% of the German population (20). Migraine sufferers often exhibit Mg^2+^ deficiency caused by stress (21). Additionally, individuals with migraines are also observed to have low Mg^2+^ levels in both the brain (22) and cerebrospinal fluid (23). Mg^2+^ may be useful as an effective first-line therapy for migraine status, especially for patients who present with lower pain intensity (24). Not only for migraines, but also for stroke, MgSO_4_ has shown promising potential as a neuroprotective therapy (25, 26). Moreover, MgSO_4_ has been found to significantly inhibit cerebral vasospasm in patients with aneurysmal subarachnoid hemorrhage (SAH) without causing severe cardiovascular complications. Continuous cisternal irrigation with MgSO_4_ solution has been shown to reduce the incidence of cerebral vasospasm in patients with SAH (27). Here, we aim to explore the relationship between Mg^2+^ supplementation and depression, as well as the involvement of microglia in depression in mice.

As one of the main cations in human cells, Mg^2+^ is concentrated in mitochondria. It is the fourth most abundant cation in the human body, following sodium, potassium, and calcium. Mg^2+^ plays a crucial role in various physiological functions and metabolic processes, including the transport of potassium and calcium ions, energy metabolism, as well as the synthesis of proteins and nucleic acids (28–30). Therefore, it is not surprising that disruptions in Mg^2+^ homeostasis are associated with a wide range of symptoms and diseases. Emerging evidence suggests that Mg^2+^ supplementation, primarily through the use of compounds like MgSO_4_ and Mg^2+^ oxide, can prevent and/or treat various disorders or diseases related to the respiratory system, reproductive system, and cardiovascular system. This beneficial effect is thought to be attributed to its anti-inflammatory, antioxidant, and antispasmodic properties (31–36). A potential link between hypomagnesemia and depression has been supported by evidence from a meta-analysis study that included 19,137 patients (37). MgSO_4_ is characterized by its easy availability, affordability, and low toxicity. However, there are limited studies demonstrating the link between Mg^2+^ supplementation and depression.

## 2 Materials and methods

### 2.1 Animal

Six-week-old male C57BL/6J mice were obtained from GemPharmatech Co., Ltd. (Jiangsu, China) and housed in a specific pathogen-free facility (temperature: 22°C ± 2°C, humidity: 55 ± 5%) at the Animal Research Center of Nanjing University of Chinese Medicine. The study was reported in accordance with ARRIVE guidelines, and all methods were carried out following relevant guidelines and regulations. The animal husbandry and experimental procedures were approved by the Animal Ethical and Welfare Committee of Nanjing University of Chinese Medicine (Approval No. 202406A042). After acclimating to the environment for 2 weeks, mice were cultured in two batches. The first batch was divided into two groups and treated as follows: exposed to a normal environment (control group, *n* = 12), while the second group was exposed to a CMS environment (CMS group, *n* = 12). The second batch was divided into five groups and treated as follows: exposed to a normal environment (control group, *n* = 12), normal saline treatment (CMS group, *n* = 12), MgSO_4_ gavage at 50 mg/kg (CMS + Mg 50 group, *n* = 12), MgSO_4_ gavage at 100 mg/kg (CMS + Mg 100 group, *n* = 12), or pioglitazone gavage at 30 mg/kg (CMS + Pi group, *n* = 12) and exposed to a CMS environment (except for control group). CMS mice were exposed to 2–3 mild stressors daily (restraint, wet/no bedding, loud noise, cage tilting, stroboscopic light, reversed light/dark cycle, food restriction, or tail suspension) after a 2-week acclimation period, continuing for 8 weeks (38). The detailed CMS methodology is shown in Supplementary Table S1. Previous studies have shown that pioglitazone could alleviate depressive-like phenotypes in mouse models induced by a high-fat diet and CMS (39, 40). Therefore, pioglitazone was selected as the positive control group. The administration methods, dosages and duration of MgSO_4_ and pioglitazone were based on previous studies (35, 41–44). After behavioral tests, a three-day rest period was provided, and then mice were sacrificed. We anesthetized the mice with 40 mg/kg sodium pentobarbital. After the completion of blood collection from the orbital venous plexus, serum and hippocampal samples were collected and immediately stored at −80°C. Whole brain tissues were preserved for immunofluorescence analysis.

### 2.2 Behavioral tests

#### 2.2.1 Open field test

Open field test (OFT) were conducted in an open field box measuring 25 × 25 × 40 cm (length, width, and height). During the 10-min OFT, the time spent and distance traveled in the center, as well as the total distance traveled, were recorded. After the 10-min OFT, mice were removed from the open field box and returned to their home cages. Subsequently, the objects and boxes were cleaned with 75% ethanol (45).

#### 2.2.2 Sucrose preference test

In brief, the mice were housed individually in a single cage for 3 days, after which they underwent an adaptation phase with normal diluent alone or containing 1% sucrose. Before sucrose preference test (SPT) test, mice were deprived of water for 24 h. Then mice were exposed to bottles containing either sucrose or water for a duration of 2 h. The total amount of liquid consumed by the mice was calculated for each bottle. The sucrose preference was calculated as the ratio of the consumed sucrose solution to the total amount of liquid consumed (46, 47).

#### 2.2.3 Forced swim test and tail suspension test

After stress, the forced swim test (FST) and tail suspension test (TST) test were performed. For details of FST, each mouse was individually placed into a cylinder filled with water (maintained at 23–25°C) to a depth of 15–20 cm. The time spent floating, swimming, and struggling was recorded using a video camera positioned directly in front of the cylinders (45, 46). For the details of the TST, each mouse was suspended individually by the end of its tail using adhesive tape in a sound-isolated room. To avoid interference, each mouse was individually partitioned for a duration of 6 min. Finally, the duration of immobility and struggling was recorded using a video camera and subsequently analyzed by trained investigators after being transferred from the computer (45, 46).

### 2.3 Determination of Mg^2+^ concentration in serum

Blood samples from the mice were centrifuged at 1,500 rpm for 15 min, and the serum was collected into clean Eppendorf tubes. Serum Mg^2+^ concentration was measured using Mg^2+^ Detection Kit (ab102506, Abcam, Cambridge, United Kingdom).

### 2.4 Determination of inflammatory cytokine

The levels of IL-1β, IL-6, and TNF-α in the serum and hippocampus were detected using enzyme-linked immunosorbent assay (ELISA) kits following the manufacturer’s instructions. The kits of IL-1β (MLB00C), IL-6 (M6000B), and TNF-α (MTA00B) were obtained from R&D Systems (Minneapolis, MN, United States).

### 2.5 Immunofluorescence staining

The mice were sacrificed, and their brains were fixed in 4% paraformaldehyde overnight. The brains were then subjected to sectioning preprocessing using standard protocols at the Analysis Center of Servivebio (Wuhan, China). The brain slices were incubated with primary antibodies, followed by a 2-h incubation with Alexa Fluor-conjugated secondary antibodies at room temperature. Nuclear staining was performed by Hoechst incubation (C1018, Beyotime, Nanjing, China) for another 30 min. The primary antibodies included Iba1 antibody (019-19741, Wako, Japan) and NF-κB p65 antibody (6956s, CST, Framingham, MA, United States). Alexa Fluor-conjugated secondary antibodies included goat anti-rabbit IgG (H + L) cross-adsorbed secondary antibody, Alexa Fluor 488 (A-11008, Invitrogen, CA, United States) as well as Alexa Fluor 555 (A-21428, Invitrogen).

### 2.6 Dextran-FITC injection for detection of BBB permeability

The mice received a tail vein injection of 200 μL dextran-fluorescein isothiocyanate (10 kDa, 20 mg/mL, FD10S, Sigma, St. Louis, United States). Ten minutes later, each mouse was anesthetized, and the brain was harvested. Next steps please refer to the methods of Immunofluorescence staining section for further details above.

### 2.7 Western blotting

Tissues or cells were rinsed with ice-cold PBS and lysed in RIPA buffer containing phenylmethylsulfonyl fluoride for 30 min. Then the lysates were centrifuged at 12,000 rpm at 4°C for 15 min. Bicinchoninic acid protein assay Kit (23005, Thermo Fisher Scientific, Wilmington, United States) was used to detect protein concentration. Proteins were separated by sodium dodecyl sulfate-polyacrylamide gel electrophoresis (SDS-PAGE) and transferred onto polyvinylidene fluoride membranes (PVDF, IPVH00010, Millipore, Billerica, United States). The membranes were blocked with 5% milk for 1 h, followed by overnight incubation with primary antibodies at 4°C, and then incubated with peroxidase-conjugated secondary antibodies for 1 h at room temperature. Primary antibodies included rabbit anti-IL-1β (ab254360, Abcam), rabbit anti-IL-6 (ab233706, Abcam, Cambridge, United Kingdom), rabbit anti-TNF-α (ab215188, Abcam), rabbit anti-p-NF-κB p65 (3033, CST), mouse anti-NF-κB p65 (6956s, CST), rabbit anti-p-IKKα/β (2697S, CST), rabbit anti-IKKα/β (R24674, Zenbio, Chengdu, China), mouse anti-p-IκBα (9246S, CST), mouse anti-IκBα (66418-1-1g, Proteintech, Wuhan, China), mouse anti-NLRP3 (68102-1-Ig, Proteintech), rabbit anti-ASC (13833S, CST), rabbit anti-cleaved caspase-1 (89332T, CST), rabbit anti-pro-caspase-1 (24232T, CST), mouse anti-β-actin (4970, CST), mouse anti-lamin B1 (66095-1-Ig, Proteintech) and rabbit anti-GAPDH (10494-1-AP, Proteintech). Secondary antibodies included goat anti-mouse IgG (H + L) HRP (YFSA01, Yfxbio, Nanjing, China), goat anti-rabbit IgG (H + L) HRP (YFSA02, Yfxbio). Relative protein levels were visualized using the Tanon 5200 chemiluminescence imaging system reagent and quantified with ImageJ software (Version 1.50b, National Institutes of Health, Bethesda, United States).

### 2.8 Flow cytometry

Before tissue collection, the brains of mice were perfused with ice-cold PBS to avoid sampling the circulating blood immune cells. The hippocampus homogenate was filtered through a 70-μm cell strainer and centrifuged again at 1,000 rpm for 3 min at 4°C. The cells were resuspended in cold PBS and centrifuged at 1,000 rpm for 3 min at 4°C. All samples were counted and diluted to a density of 1–2 × 10^5^/mL, and then labeled using a Live/Dead kit for 30 min, centrifuged at 1,000 rpm for 3 min at 4°C. The cells were resuspended in 100 μL PBS buffer and blocked with anti-CD16/32 (553142, BD Biosciences, New Jersey, United States) for 10 min. The cells were incubated with the antibodies for flow cytometry according to the manufacturers’ protocols for 30 min at 4°C. The following antibodies were used in the FACS analysis: CD45-BV510 (561487, BD Biosciences, Franklin Lakes, United States), CD11b-Alexa 488 (557672, BD Biosciences). Cells were detected by a BD Aria III cytometer, and the data were analyzed by FlowJo software.

### 2.9 Quantitative real-time PCR

Total mRNA was isolated from tissues or cells using TRIzol reagent (#15596026, Invitrogen, Carlsbad, CA) according to the manufacturer’s instructions. Total RNA (1 μg) was reverse-transcribed using the HiScript II select qRT supermix (R222-01, Vazyme, Jiangsu, China). Quantitative real-time PCR was performed using gene specific primer sets and SYBR Green (Vazyme) on a quantitative real-time PCR (qRT-PCR) detection system (Bio-Rad, California, United States). All primers were designed by ourselves and synthesized in the Generay Biotech Co., Ltd. (Shanghai, China).

### 2.10 Statistics

Data were shown as mean ± SEM. All the data were examined for normality with the Skew test. Two-tailed Student’s *t*-test was used for comparisons between two groups, and data were analyzed statistically using one-way ANOVA for comparisons of more than two groups with single factor variance. Statistical analyses were performed with GraphPad Prism Software (GraphPad Software, San Diego, United States). Value of *p* < 0.05 was considered statistically significant (^*^p < 0.05, ^**^p < 0.01, and ^***^p < 0.001).

## 3 Result

### 3.1 CMS induced depressive-like behavior as well as hypomagnesemia in mice

**Figure 1:**
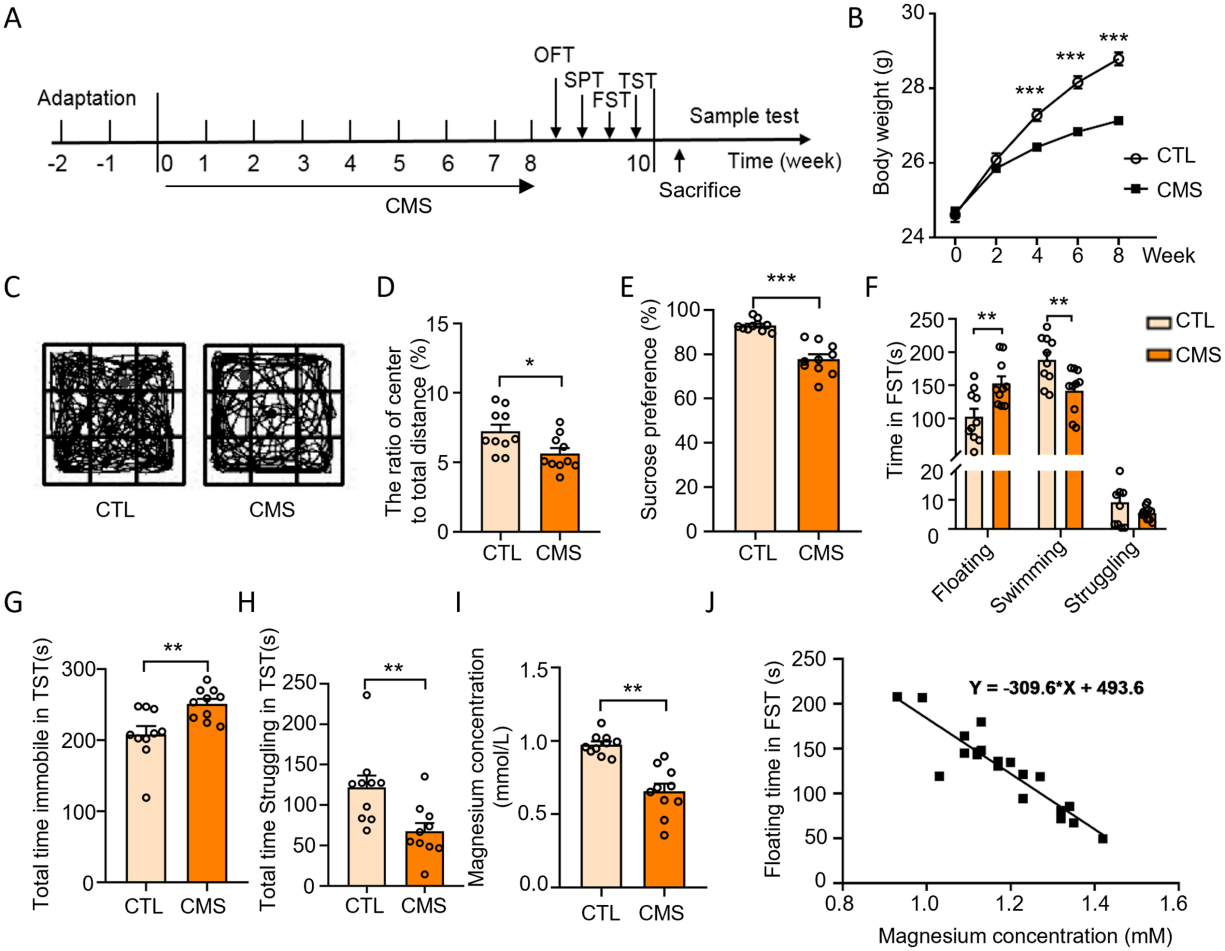
CMS induced depressive-like behavior as well as hypomagnesemia in mice. **(A)** Schematic diagram illustrating the experimental design depicting mice subjected to CMS and subsequent behavioral tests. **(B)** Body weight of mice was recorded during CMS handling (*n* = 12). **(C,D)** Behavioral test and representative state images of mice in OFT (*n* = 10). **(E)** Sucrose preference performance of mice in SPT (*n* = 10). **(F)** Time spent floating, swimming, and struggling in FST (*n* = 10). **(G)** Total time immobile in TST (*n* = 10). **(H)** Total time struggling in TST (*n* = 10). **(I)** Serum Mg^2+^ levels in mice (*n* = 10). **(J)** Regression analysis of serum Mg^2+^ concentration and floating time of mice in FST. CTL, control group; CMS, CMS-induced group. Data are expressed as mean ± SEM, ^*^p < 0.05, ^**^p < 0.01, and ^***^p < 0.001.

Mice were suffered with CMS for 8 weeks. At the end of stress, depressive-like behaviors were assessed with OFT, SPT, FST and TST (Figure 1A). The body weight of CMS mice exhibited a significant decrease compared to the control group (Figure 1B). CMS mice demonstrated a decrease in central area activity time, the ratio of the distance of central movement to the total distance in the OFT (Figures 1C,D; Supplementary Figure S1) and a reduced sucrose preference in the SPT (Figure 1E) compared with control group. The CMS procedure led to an increased floating time of mice in the FST (Figure 1F), as well as the immobile time in the TST (Figure 1G). Moreover, it resulted in a decrease in the swimming time and struggling time in the FST and TST, respectively (Figures 1F,H). Taken together, the mice developed depressive-like behaviors after experiencing a protocol of CMS. Notably, we found that CMS mice had a lower Mg^2+^ concentration in the serum when compared to the control group (Figure 1I) and the Mg^2+^ concentration was negatively correlated with the floating time of FST in mice (Figure 1J). These results demonstrated CMS mice displayed depressive-like behaviors accompanied by hypomagnesemia.

### 3.2 Depressive-like mice exhibited hippocampal inflammatory response with microglia activation and BBB impairment

**Figure 2:**
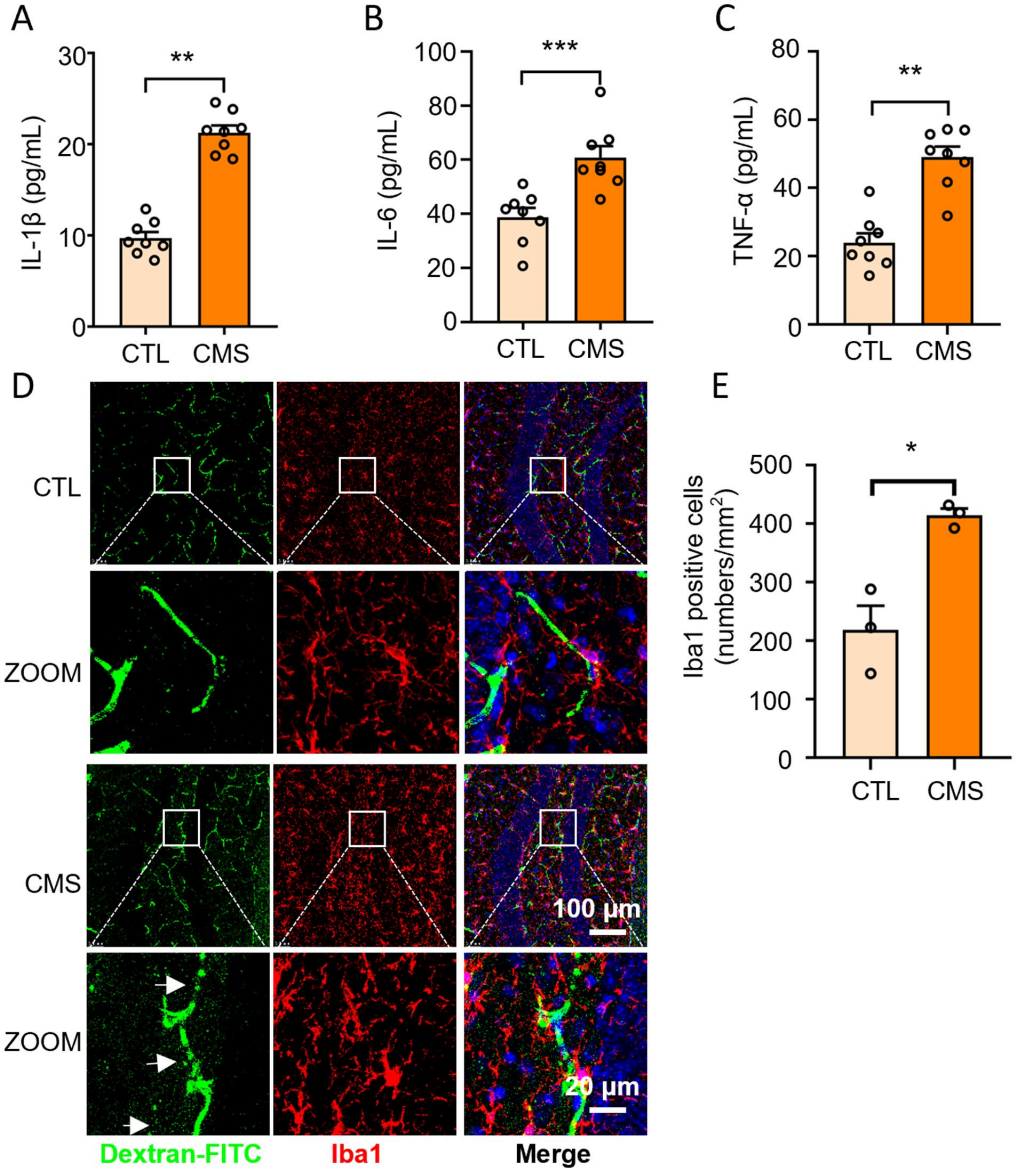
Depressive-like mice exhibited increased inflammatory level in serum, activated microglia and BBB impairment. **(A)** Serum IL-1β levels (*n* = 8). **(B)** Serum IL-6 levels (*n* = 8). **(C)** Serum TNF-α levels (*n* = 8). **(D)** Representative confocal images labeled with Iba1 and dextran-FITC. The white arrows refer to the obvious green fluorescence that permeates outside the blood vessels. **(E)** Quantification of the number of Iba1-positive microglia (*n* = 3). CTL, control group; CMS, CMS-induced group. Data are presented as mean ± SEM; ^*^p < 0.05, ^**^p < 0.01, and ^***^p < 0.001.

The mice subjected to CMS procedure exhibited up-regulation of IL-6, IL-1β, and TNF-α levels in serum compared to the control group (Figures 2A–C). Next, we explored whether CMS developed neuroinflammatory response by counting the number of microglia and observing the morphology of microglia (positive for Iba1). Microglia, a type of glial cells equivalent to macrophages in the brain and spinal cord, are considered as the first and most crucial line of immune defense in the central nervous system. Immunofluorescence results revealed a significant increase in the number of microglia in CMS mice, accompanied by enlarged cell bodies and reduced branches compared with control group (Figures 2D,E). The occurrence of neuroinflammatory response not only displayed activation of microglia, but is often accompanied by damage to the blood-brain barrier (BBB). Haruwaka et al. (48) demonstrated that during sustained inflammation, microglia phagocytosed astrocytic end-feet, impairing the function of BBB. Here, we also found that CMS-induced neuroinflammation and compromised the permeability of the BBB by injecting 10 kDa dextran through the tail vein (Figure 2D). These results suggested that CMS promoted the secretion of inflammatory factor and microglia activation, which may be harm to the BBB.

### 3.3 MgSO_4_ and pioglitazone inhibit hippocampal neuroinflammation in CMS mice

**Figure 3:**
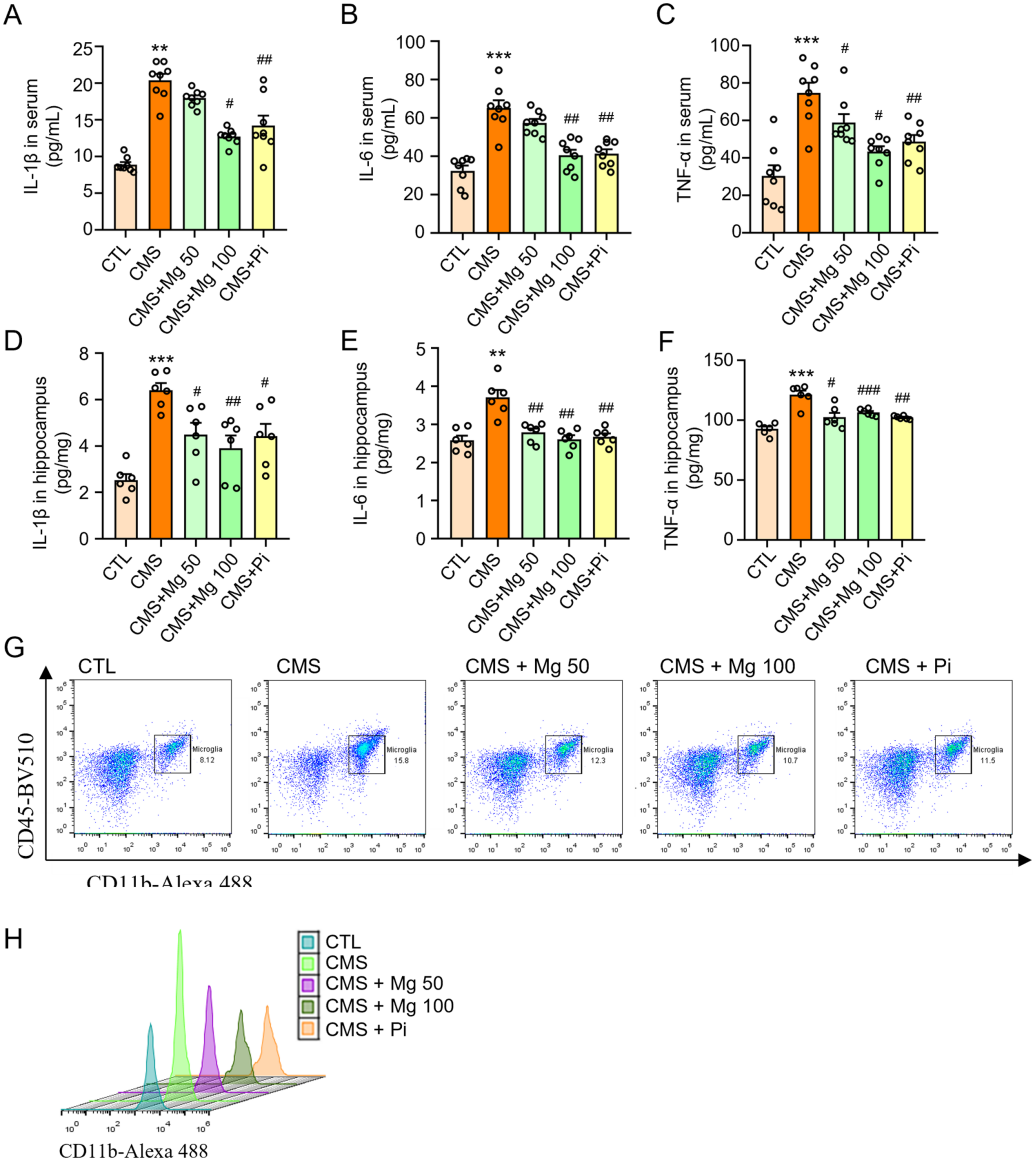
MgSO_4_ and pioglitazone inhibit hippocampal neuroinflammation in CMS mice. **(A)** Serum IL-1β levels (*n* = 8). **(B)** Serum IL-6 levels (*n* = 8). **(C)** Serum TNF-α levels (*n* = 8). **(D)** IL-1β levels in the hippocampus (*n* = 6). **(E)** IL-6 levels in the hippocampus (*n* = 6). **(F)** TNF-α levels in the hippocampus (*n* = 6). **(G,H)** Microglia (CD45 low CD11b^+^) were detected by flow cytometry and shown with dot and peak plots (*n* = 3). CTL, control group; CMS, CMS-induced group; CMS + Mg 50, 50 mg/kg MgSO_4_ gavage group; CMS + Mg 100, 100 mg/kg MgSO_4_ gavage group; CMS + Pi, 30 mg/kg pioglitazone gavage group. Data are presented as mean ± SEM. ^*^Indicated significant difference (^*^p < 0.05, ^**^p < 0.01, and ^***^p < 0.001) between control and CMS groups. ^#^Represented a significant difference (^#^*p* < 0.05, ^##^*p* < 0.01, and ^###^*p* < 0.001) between CMS and CMS + Mg 50 groups, CMS + Mg 100 groups or CMS + Pi groups.

We have found that depressive-like mice are accompanied by a decrease of Mg^2+^ concentrations in serum. Next, we further explore whether Mg^2+^ supplementation improves the neuroinflammatory response in mice. We observed that oral treatment with MgSO_4_ (50 or 100 mg/kg) significantly reduced the cytokine levels of IL-1β, IL-6, and TNF-α in the serum (Figures 3A–C) as well as in the hippocampus (Figures 3D–F) compared with CMS mice. Moreover, MgSO_4_ treatment reduced the proportion of Iba1^+^ microglia in the hippocampus of CMS mice (Figures 3G,H). Similarly, pioglitazone significantly decreased the levels of IL-1β, IL-6, and TNF-α, and the number of Iba1^+^ microglia in the hippocampus of CMS mice (Figure 3). These results suggest that treatment with MgSO_4_ and pioglitazone effectively inhibits hippocampal neuroinflammation in CMS mice.

### 3.4 MgSO_4_ and pioglitazone inhibit microglial activation via IKK/NF-kB and NLRP3 inflammasome signaling pathways

**Figure 4:**
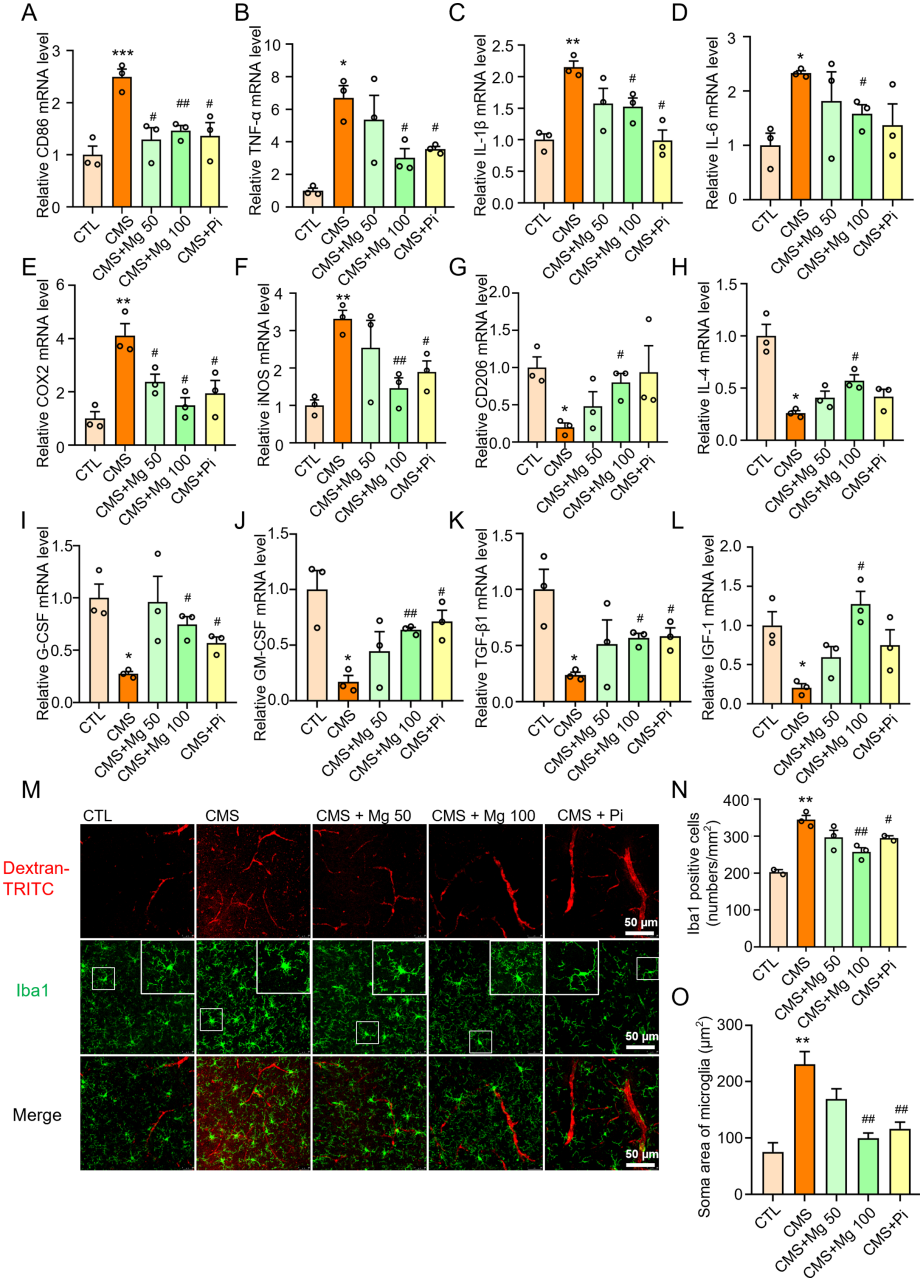
MgSO_4_ and pioglitazone inhibit the activation of microglia and improve BBB damage. **(A–F)** The mRNA levels of M1 markers in the hippocampus were detected by qRT-PCR, including CD86, TNF-α, IL-1β, IL-6, COX-2, and iNOS (*n* = 3). **(G–L)** The mRNA levels of M2 markers in the hippocampus were detected by qRT-PCR, including CD206, IL-4, G-CSF, GM-CSF, TGF-β1, and IGF-1 (*n* = 3). **(M)** Representative confocal images labeled with Iba1 and dextran-TRITC. **(N)** Quantification of the number of Iba1-positive microglia (*n* = 3). **(O)** Quantification of the soma area of Iba1-positive microglia (*n* = 3). CTL, control group; CMS, CMS-induced group; CMS + Mg 50, 50 mg/kg MgSO_4_ gavage group; CMS + Mg 100, 100 mg/kg MgSO_4_ gavage group; CMS + Pi, 30 mg/kg pioglitazone gavage group. Data are presented as mean ± SEM. ^*^Indicated significant difference (^*^p < 0.05, ^**^p < 0.01, and ^***^p < 0.001) between control and CMS groups. ^#^Represented a significant difference (^#^*p* < 0.05, ^##^*p* < 0.01, and ^###^*p* < 0.001) between CMS and CMS + Mg 50 groups, CMS + Mg 100 groups or CMS + Pi groups.

Microglial activation is characterized not only by an increase in cell number, but also by morphological changes, often involving the M1-to-M2 shift phenotype (15, 49). Compared to the control group, CMS significantly upregulates the mRNA levels of M1 markers in the hippocampus of mice, including CD86, TNF-α, IL-1β, IL-6, COX-2, and iNOS, while treatment with MgSO_4_ and pioglitazone reverses the mRNA expression of M1 markers (Figures 4A–F). In addition, CMS treatment significantly decreases the mRNA levels of M2 markers, including CD206, IL-4, G-CSF, GM-CSF, TGF-β1, and IGF-1. MgSO_4_ and pioglitazone treatment increased the expression of M2 markers (Figures 4G–L). *In vitro*, MgSO_4_ also significantly reversed the M1-to-M2 transition induced by lipopolysaccharide (LPS) in BV2 microglia. Compared to the control group, LPS significantly upregulated the mRNA levels of M1 markers and downregulated the M2 markers in BV2 cells, while treatment with MgSO_4_ and pioglitazone reverses these changes (Supplementary Figures S2A–J). Therefore, we determined that MgSO_4_ has a specific effect on microglia. Moreover, immunofluorescence results of microglia showed that after CMS induction, the size of cell bodies increased and the ramification of distal branches decreased, and MgSO_4_ and pioglitazone reversed these morphological transformations of microglia (Figures 4M–O). Notably, MgSO_4_ and pioglitazone also alleviated CMS-induced dextran leakage of BBB (Figure 4M). Therefore, MgSO_4_ treatment could inhibit microglia activation and BBB damage induced by CMS.

**Figure 5:**
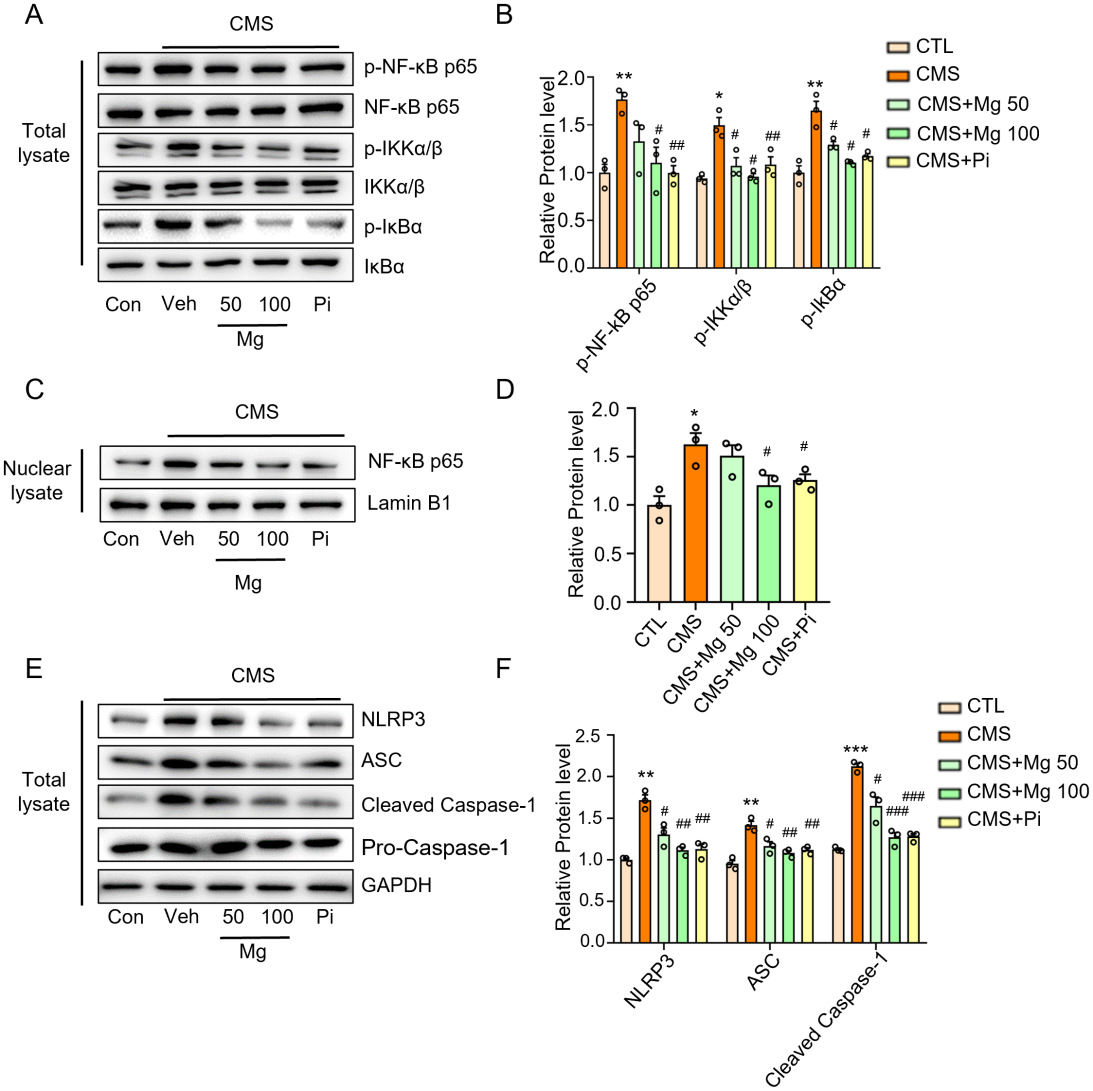
MgSO_4_ and pioglitazone inhibit microglial activation via IKK/NF-kB and NLRP3 inflammasome signaling pathways. **(A)** Protein expression detected by western blotting including p-NF-κB p65, NF-κB p65, p-IKKα/β, IKKα/β, p-IκBα, and IκBα. **(B)** Densitometry analysis of p-NF-κB p65, p-IKKα/β, and p-IκBα (*n* = 3). **(C)** Protein expression detected by western blotting including nuclear NF-κB p65 and lamin B1. **(D)** Densitometry analysis of nuclear NF-κB p65 (*n* = 3). **(E)** Protein expression detected by western blotting including NLRP3, ASC, caspase-1, pro-caspase-1, and GAPDH. **(F)** Densitometry analysis of NLRP3, ASC, and pro-caspase-1 (*n* = 3). CTL, control group; CMS, CMS-induced group; CMS + Mg 50, 50 mg/kg MgSO_4_ gavage group; CMS + Mg 100, 100 mg/kg MgSO_4_ gavage group; CMS + Pi, 30 mg/kg pioglitazone gavage group. Data are presented as mean ± SEM. ^*^Indicated significant difference (^*^p < 0.05, ^**^p < 0.01, and ^***^p < 0.001) between control and CMS groups. ^#^Represented a significant difference (^#^*p* < 0.05, ^##^*p* < 0.01, and ^###^*p* < 0.001) between CMS and CMS + Mg 50 groups, CMS + Mg 100 groups or CMS + Pi groups.

NF-κB signaling and NLRP3 inflammasome are important transduction signals of inflammatory reaction in mammals (50, 51). The CMS-induced mice mentioned above exhibited higher levels of IL-1β, IL-6, and TNF-α in both serum and hippocampus compared to the control mice (Figures 3A–E). Here, we observed increased protein levels of p-NF-κB p65, p-IKKα/β, p-IκBα, nuclear NF-κB, as well as NLRP3, ASC, and pro-caspase-1 in the hippocampus of CMS mice. Similarly, nuclear proteins were extracted, revealing that CMS facilitated the translocation of NF-κB into the nucleus. This observation indicates that CMS promotes the activation of NF-κB signaling and the NLRP3 inflammasome in the hippocampus (Figures 5A–F). MgSO_4_ and pioglitazone reduced p-NF-κB p65, p-IκBα and p-IKKα/β expression, and inhibited NF-κB entry into the nucleus (Figures 5A–D). Similarly, *in vitro*, NF-κB (green fluorescence) entered the nucleus in LPS-induced BV2 cells compared to the control group, while MgSO_4_ significantly reversed the phenomenon of NF-κB entering the nucleus (Supplementary Figure S2K). Moreover, MgSO_4_ and pioglitazone inhibited the activation of the NLRP3 inflammasome (Figures 5E,F) in the hippocampus of CMS mice, which was consistent with the alleviation of hippocampal neuroinflammation and BBB damage mentioned above. These results suggested that MgSO_4_ and pioglitazone may inhibit the activation of microglia by inhibiting NF-κB signaling and NLRP3 inflammasome to alleviate hippocampal neuroinflammation and repair BBB damage.

### 3.5 MgSO_4_ and pioglitazone alleviated the depressive-like behavior of CMS mice

**Figure 6:**
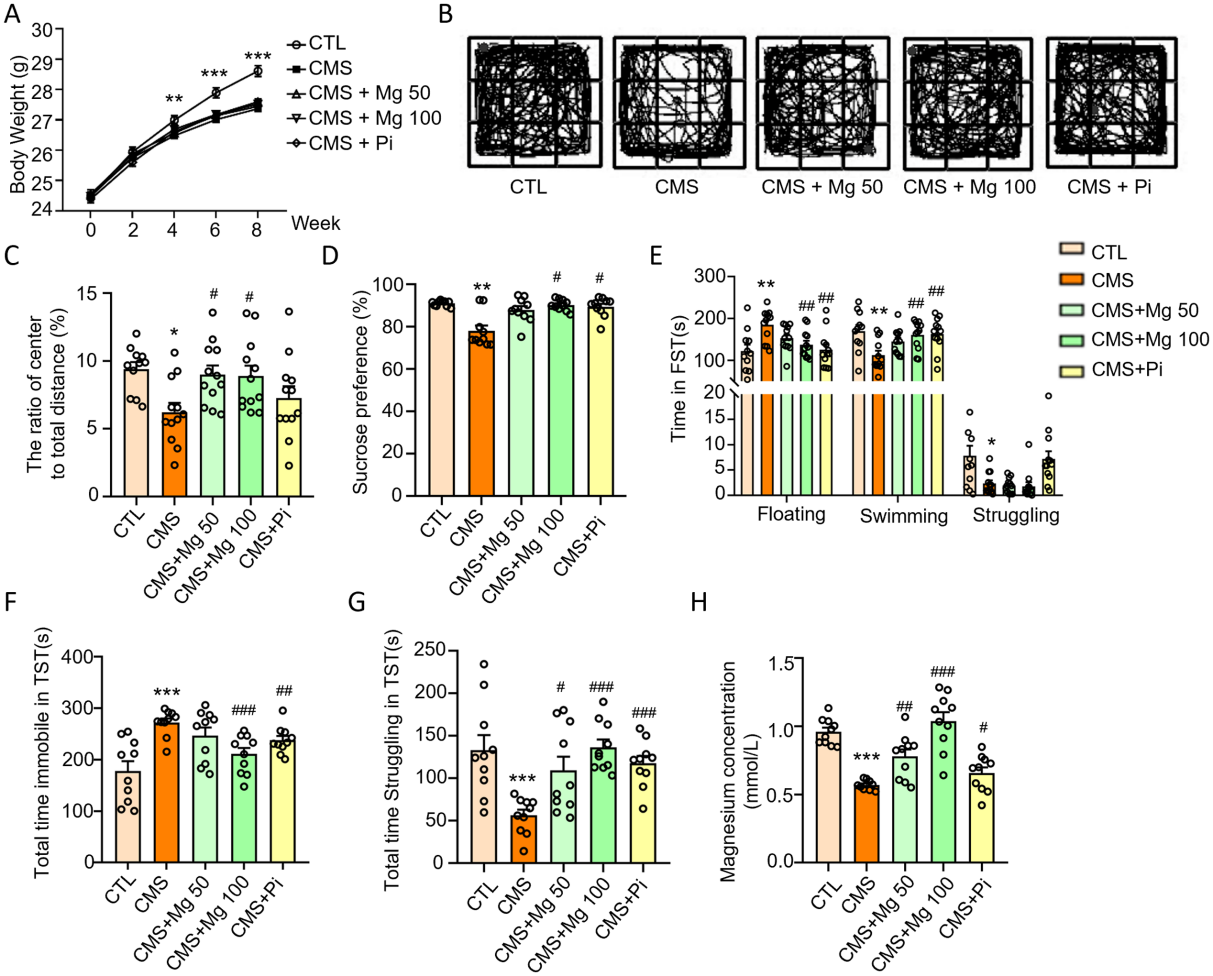
MgSO_4_ and pioglitazone alleviated the depressive-like behavior of CMS mice. **(A)** Body weight of mice (*n* = 11–12). **(B,C)** The ratio of center to total distance and representative state images of mice in OFT (*n* = 11–12). **(D)** SPT performance of mice (*n* = 10). **(E)** Time spent floating, swimming, and struggling in FST (*n* = 11–12). **(F)** Total time immobile in TST (*n* = 10). **(G)** Total time struggling in TST. **(H)** Serum Mg^2+^ levels in mice (*n* = 10). CTL, control group; CMS, CMS-induced group; CMS + Mg 50, 50 mg/kg MgSO_4_ gavage group; CMS + Mg 100, 100 mg/kg MgSO_4_ gavage group; CMS + Pi, 30 mg/kg pioglitazone gavage group. Data are expressed as mean ± SEM. ^*^Indicated significant difference (^*^p < 0.05, ^**^p < 0.01, and ^***^p < 0.001) between control and CMS groups. ^#^Represented a significant difference (^#^*p* < 0.05, ^##^*p* < 0.01, and ^###^*p* < 0.001) between CMS and CMS + Mg 50 groups, CMS + Mg 100 groups or CMS + Pi groups.

To further investigate the effects of Mg^2+^ supplementation on CMS-induced depressive-like behaviors in mice, we performed OFT, SPT, FST and TST experiments. MgSO_4_ were unable to restore the body weight of CMS mice (Figure 6A), but increased the central area activity time, distance and the ratio of the distance of central movement to the total distance in the OFT (Figures 6B,C; Supplementary Figure S3) and sucrose preference in the SPT (Figure 6D) in CMS mice. Moreover, Both MgSO_4_ and pioglitazone reduced the floating time of CMS mice in the FST (Figure 6E), as well as the immobile time in the TST (Figure 6F), while increasing swimming time and struggling time in the FST and TST, respectively (Figures 6E,G). Notably, MgSO₄ significantly alleviated serum Mg^2+^ levels in CMS-induced mice (Figure 6H).

**Figure 7:**
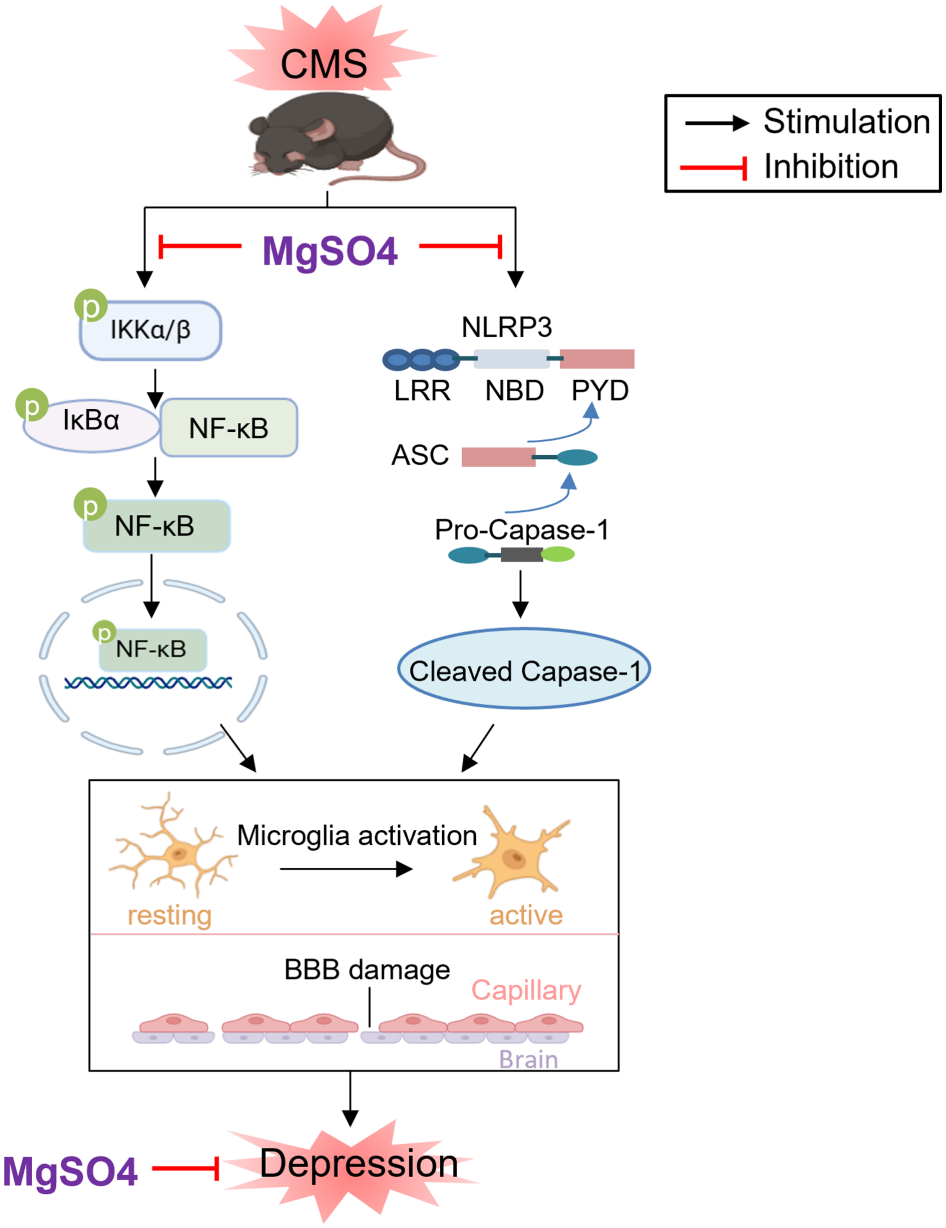
Schematic diagram of the neuroprotective effect of MgSO_4_: MgSO_4_ was found to suppress IKK/NF-κB and NLRP3 inflammasome signaling pathways, leading to the improvement of hippocampal neuroinflammation and BBB damage. This suppression ultimately resulted in the amelioration of depressive-like behaviors in mice exposed to CMS. Created with BioRender.com.

Therefore, the depressive-like behaviors of mice experienced CMS could be alleviated through treatment with MgSO_4_ and pioglitazone. The mechanism by which MgSO_4_ improves depressive-like behavior in CMS mice is illustrated in Figure 7.

## 4 Discussion

In this study, we observed that CMS mice exhibited depressive-like behaviors along with a reduction in serum Mg^2+^ concentration. Furthermore, CMS promoted the hippocampal neuroinflammation characterized by microglia activation and BBB damage. MgSO_4_ prevented hippocampal neuroinflammation, BBB damage and microglia activation, and improved depressive-like behaviors in CMS-induced mice.

The detection of Mg^2+^ concentration is not yet a routine procedure in clinical practice. However, it has been observed that many patients with the disease exhibit hypomagnesemia, which may have a significant correlation with the patient’s symptoms and disease progression. Moreover, multiple studies have demonstrated the beneficial effects of Mg^2+^ supplementation for a variety of diseases. For instance, nebulized inhalation and intravenous injection of MgSO_4_ have shown positive clinical outcomes in the treatment of childhood asthma (52, 53). In the case of acute asthmatic adults, MgSO_4_ supplement is reported to improve pulmonary symptoms and decrease hospitalization rates among patients (54). In addition, Mg^2+^ is considered an effective neuroprotective agent in acute stroke (25). Meanwhile, it has been observed that mice fed with a low Mg^2+^ diet exhibit heightened depression- and anxiety-related behaviors (55). In a clinical trial, short-term MgSO_4_ treatment was found to be ineffective in alleviating depressive symptoms in patients with depression. However, for the long-term treatment, MgSO_4_ exactly improved depressive symptoms with elevating Mg^2+^ concentration in serum (56). Here, we found that Mg^2+^ supplementation improved depressive-like behaviors in mice induced by CMS. These results provide further support for the notion that maintaining Mg^2+^ homeostasis could play a crucial role in preventing depression.

It has been demonstrated that damage to the BBB can increase the risk of developing depression (57, 58). Here, BBB integrity of mice was visualized by dextran-FITC injection, and BBB damage was observed in mice exposed to CMS. Moreover, it has been revealed that overactivated microglia phagocytose astrocytic end-feet and impair BBB function in mice with sustained systemic inflammation (48). Microglial hyperactivation, characterized by an increased number of microglia, enlarged cell bodies, and reduced branches, was observed in the hippocampus of CMS mice, potentially contributing to BBB damage.

Neuroinflammation is an indispensable element in the process of depression. In a rat model of eclampsia, MgSO_4_ treatment was found to decrease brain water content, reduce neuronal death in the hippocampal area, and inhibit the expression of IL-1β and TNF-α. These findings suggest that MgSO_4_ may exert neuroprotective effects through its anti-inflammatory properties (59). NF-κB signaling and NLRP3 inflammasome play crucial roles as transduction signals in inflammatory reactions, particularly in regulating the expression of IL-1β, TNF-α, and IL-6 (50, 51). It has been reported that increased blood pressure due to dietary Mg^2+^ depletion leads to elevated NLRP3 and IL-1β production in mice (60). Additionally, short-term dietary magnesium deficiency in rats causes the upregulation of neutral sphingomyelinase (N-SMase), which may activate transcription factors (e.g., p53, NF-κB) and promote cytokine release in cardiovascular tissues and cells (61). Our study showed that Mg^2+^ supplementation suppressed the hyperactivation of microglia and alleviated BBB damage in mice exposed to CMS, possibly by inhibiting IKK/NF-κB and NLRP3 inflammasome signaling pathways. These results revealed the mechanisms of Mg^2+^ supplementation in reducing the risk of depression. Although we demonstrated that the protective effect of Mg^2+^ supplementation on depressed mice may be mainly through the protection of the BBB and inhibition of microglial cell activation, the effect of Mg^2+^ supplementation on both hippocampal neurogenesis and neuronal circuits in mice cannot be ruled out (45, 62). Further research is required to substantiate these effects in future studies.

This study still has some limitations. Despite measuring post-treatment serum Mg^2+^ levels (Figure 6H), the lack of time-dependent MgSO₄ pharmacokinetic profiling, which includes a comprehensive pharmacokinetic profile (absorption, distribution, metabolism, and excretion) and verification of therapeutic-level maintenance at critical time points. Moreover, although female mice exhibit higher depression susceptibility during hormonally dynamic phases (e.g., postpartum, menopause) (63), we exclusively used male mice to minimize confounding effects from cyclic estrogen and progesterone fluctuations on neurochemical and behavioral outcomes (64). Future studies will address these gaps by integrating pharmacokinetic analyses and sex-specific investigations to enhance translational relevance.

## 5 Conclusion

In summary, our findings suggest that MgSO_4_ has the potential to inhibit microglial activation, improve BBB damage, and mitigate depressive-like behaviors in mice subjected to CMS, likely via suppression of the IKK/NF-κB and NLRP3 inflammasome signaling pathways. Therefore, these results may have implications for the development of interventions aimed at promoting a healthy diet supplemented with Mg^2+^ to prevent depression.

## Data Availability

The original contributions presented in the study are included in the article/Supplementary material, further inquiries can be directed to the corresponding authors.
