# Supplementary material for: MgSO4 alleviates hippocampal neuroinflammation and BBB damage to resist CMS-induced depression
Source: Front Nutr. 2025 Mar 26;12:1470505. doi: 10.3389/fnut.2025.1470505 (PMC11979798; doi:10.3389/fnut.2025.1470505)
Supplement: Supplementary file 1 [file Data_Sheet_1.docx]

**Supporting Information for**

**Original article**

**MgSO_4_ alleviates hippocampal neuroinflammation and BBB damage to resist CMS-induced depression**

Qiaona Wang ^1^, Yuefeng Hu ^2, 3^, Liyun Hu ^3^, Fan Li ^3^, Yizhu Zhang ^2^, Yunfa Qiao ^1 *^, Chuanfeng Tang ^2 *^, Renlei Wang ^1, 4 *^

1. School of Ecology and Applied Meteorology, Nanjing University of Information Science & Technology, Nanjing 210044, People's Republic of China.

2. State Key Laboratory on Technologies for Chinese Medicine Pharmaceutical Process Control and Intelligent Manufacture, Nanjing University of Chinese Medicine, Nanjing 210023, People's Republic of China.

3. School of Food Science and Pharmaceutical Engineering, Nanjing Normal University, Nanjing 210023, People's Republic of China.

4. Biology Department, Jiangsu Second Normal University, No. 77 Beijing West Road, Nanjing 210013, People's Republic of China.

^*^ Corresponding author: Renlei Wang, Yunfa Qiao, Chuanfeng Tang

E-mail address: wrl3501988@163.com (R.W.); qiaoyunfa@nuist.edu.cn (Y.Q.); 300645@njucm.edu.cn (C.T.)

**This file includes:**

**1. Supporting methods**

**2. Supporting figures (Figures S1–S3)**

**3. Supporting tables (Table S1)**

**1. Supporting methods**

**1.1 Cell culture**

The BV2 cell line was purchased from Pricella (CL-0493, Pricella, Wuhan, China) and culture at 37°C in a humidified atmosphere containing 5% CO^2^. For different level Mg^2+^ experiment, customized culture media without Mg2+ are obtained from Pricella and different level of MgSO_4_ was added as required for individual experiments.

**1.2 drug management**

To investigate the effect of Mg2+ on neuroinflammation, BV2 cells were divided into the following five groups: (a) CTL: control group; (b) LPS group: cells were incubated in medium containing 200 ng/mL LPS for 24h. (c) LPS + Mg 0.5: cells were incubated in medium containing 0.5 mM MgSO_4_ and 200 ng/mL LPS for 24 h; (d) LPS + Mg 1: cells were incubated in medium containing 1 mM MgSO_4_ and 200 ng/mL LPS for 24 h; (e) LPS + Pi: cells were incubated in medium containing 10 μM pioglitazone and 200 ng/mL LPS for 24 h. The administration methods, dosages and length of MgSO4 and pioglitazone were based on previous studies [1-4].

**2. Supporting figures (Figures S1–S3)**


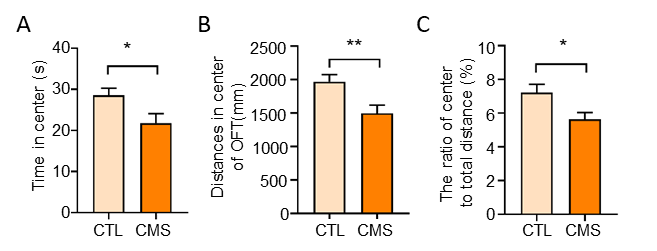


Figure S1. The time (A) and distance (B) in center traveled by mice in the OFT. Data are expressed as mean ± SEM, **P* < 0.05, ***P* < 0.01.


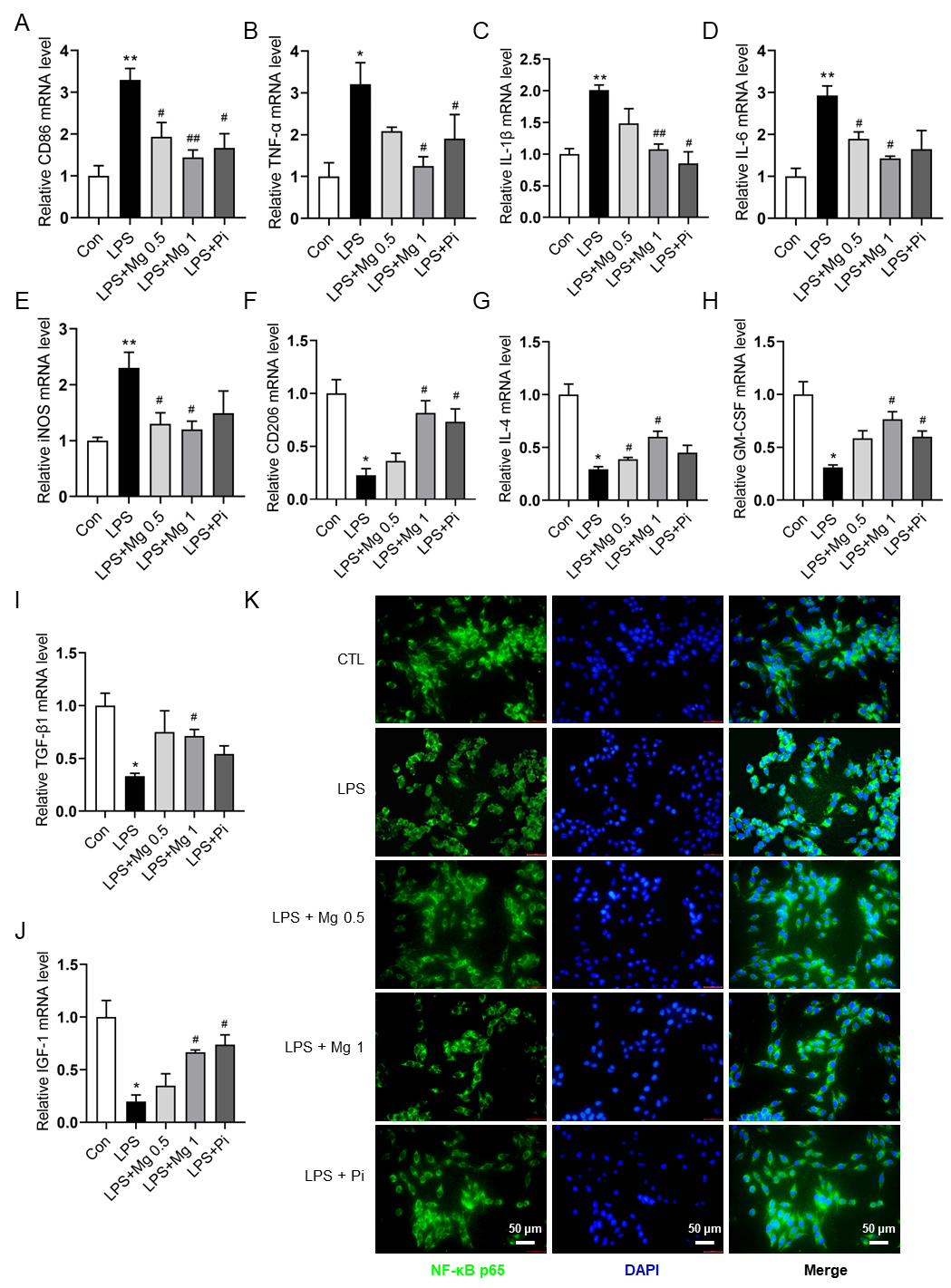


Figure S2. MgSO4 and pioglitazone inhibit the activation of BV2 microglia cells and NF-κB signaling pathway. (A-E) The mRNA levels of M1 markers in the BV2 cells were detected by qRT-PCR, including CD86, TNF-α, IL-1β, IL-6, COX-2, and iNOS. (F-J) The mRNA levels of M2 markers in the BV2 cells were detected by qRT-PCR, including CD206, IL-4, GM-CSF, TGF-β1, and IGF-1. (K) Representative confocal images labeled with NF-κB and DAPI. CTL, control group; LPS, LPS (1 μg/mL) treated group; LPS + Mg 0.5, 0.5 mM MgSO4 treatment group; LPS + Mg 1, 1 mM MgSO4 treatment group; LPS + Pi, 10 μM pioglitazone treatment group. * Indicated significant difference (**P* < 0.05, ** *P* < 0.01, *** *P* < 0.001) between control and LPS groups. # represented a significant difference (^#^*P* < 0.05, ^##^*P* < 0.01, ^###^*P* < 0.001) between LPS and LPS +Mg 0.5 groups, LPS +Mg 1 groups or LPS +Pi groups.


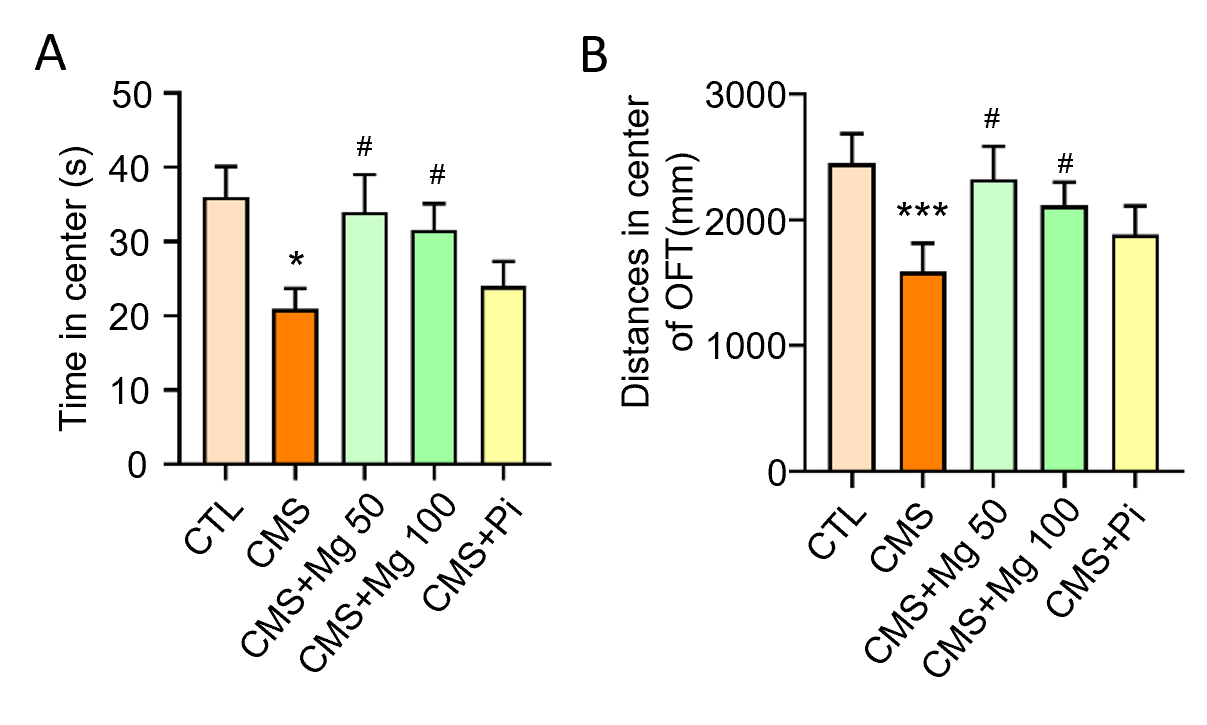


Figure S3. The time (A) and distance (B) in center traveled by mice in the OFT. Data are presented as mean ± SEM; *Indicated significant difference (**P* < 0.05, ***P* < 0.01, ****P* < 0.001) between control and CMS groups. ^#^ represented a significant difference (^#^*P* < 0.05, ^##^*P* < 0.01, ^###^*P* < 0.001) between CMS and CMS + Mg 50 groups, CMS + Mg 100 groups or CMS + Pi groups.

**3. Supporting tables (Table S1)**

|  | Monday | Tuesday | Wednesday | Thursday | Friday | Saturday | Sunday |
| --- | --- | --- | --- | --- | --- | --- | --- |
| Week1 | 10:00-12:00  Loud noise | 10:00-11:00  Restraint | 10:00-12:00  Stroboscopic  light | 11:00-13:00  Loud noise | 10:00-12:00  Stroboscopic light | Inversion  light/dark cycle | Inversion  light/dark  cycle |
|  | 15:00-15:30  Tail suspension | 13-15h  Loud noise | 15:00-16:00 Restraint  stress | 14:30-16:30  Stroboscopic  light | 16:00-17:00 Restraint stress |  |  |
|  | 20:00-21:00 Stroboscopic light | 16:00-22:00  Food restriction | 18:00-20:00  Loud noise | 19:00-9:30 Wet cage | 19:00-10:00  Wet cage |  |  |
| Week2 | 10:00-12:00  Loud noise | Inversion  light/dark cycle | Inversion  light/dark cycle | 10:00-12:00  Loud noise | 10:00-11:00  Restraint stress | 11:00-13:00 Stroboscopic  light | 10:00-12:00  Loud noise |
|  | 15:00-17:00 Stroboscopic  light |  |  | 14:00-16:00 Stroboscopic  light | 14:00-16:00 Loud noise | 15:00-16:00 Restraint stress | 14:00-15:00 Restraint stress |
|  | 19:00-9:30  Wet cage |  |  | 19:00-9:30  Wet cage | 17:00-23:00  Food restriction | 19:00-10:00  Tilted cage | 18:00-20:00 Stroboscopic  light |
| Week3 | 10:00-12:00  Loud noise | 11-13h  Loud noise | 10:30-12:30h Stroboscopic  light | 10:00-12:00  Loud noise | 10:00-11:00  Restraint | Inversion  light/dark cycle | Inversion  light/dark cycle |
|  | 14:30-15:30h Restraint stress | 15:00-17:00 Stroboscopic light | 15:00-17:00  Loud noise | 14:00-16:00 Stroboscopic light | 13-15h  Loud noise |  |  |
|  | 19:00-20:30h Wet cage | 20；00-10;00  Tilted cage | 19:30-21:00 Restraint stress | 17:00-9:30  Tilted cage | 16:00-22:00  Food restriction |  |  |
| Week4 | 11:00-13:00  Loud noise | 10:00-12:00  Stroboscopic  light | 10:00-12:00  Stroboscopic  light | 10:30-12:30h Stroboscopic light | 10:00-12:00  Loud noise | Inversion  light/dark cycle | Inversion  light/dark cycle |
|  | 15:00-17:00 Stroboscopic light | 14:00-16:00 Loud noise | 15:30-16:30 Restraint stress | 15:30 -16:30  Restraint stress | 15:00-15:30  Tail suspension |  |  |
|  | 19:30-20:30 Restraint stress | 19:30-9:30 Tilted cage | 18:00-20:00  Loud noise | 21:00 -9:00  Tilited cage | 20:00-21:00 Stroboscopic light |  |  |
| Week5 | 11:00-13:00  Stroboscopic light | Inversion  light/dark cycle | Inversion  light/dark cycle | 10:00-12:00  Loud noise | 10:30-12:30 Stroboscopic light | 10:30-12:00  Stroboscopic  light | 10:00-12:00  Loud noise |
|  | 15:00-17:00  Loud noise |  |  | 15:30 -16:30  Restraint stress | 15:00-17:00  Loud noise | 16:00-17:00 Restraint stress | 15:30 -16:30  Restraint stress |
|  | 20:00-21:00 Restraint stress |  |  | 21:00-9:30  Tilted cage |  | 19:00-21:00  Loud noise | 21:00-9:30  Tilted cage |
| Week6 | 11:00-13:00  Stroboscopic light | 9:30-11:30 Loud noise | Inversion  light/dark cycle | Inversion  light/dark cycle | 10:30-12:30 Stroboscopic light | 9:30-11:30 Loud noise | 10:00-12:00  Stroboscopic  light |
|  | 15:00-17:00  Loud noise | 15:30-17:30 Stroboscopic light |  |  | 15:00-17:00  Loud noise | 15:30-17:30 Stroboscopic light | 16:00-17:00 Restraint stress |
|  | 20:00-21:00 Restraint stress | 20:30-9:30 Tilted cage |  |  |  | 20:30-9:30 Tilted cage | 19:00-21:00  Loud noise |
| Week7 | 10:00-12:00  Loud noise | 9:30-11:30 Loud noise | 10:00-12:00  Stroboscopic  light | 10:00-12:00  Loud noise | 10:30-12:30 Stroboscopic light | Inversion  light/dark cycle | Inversion  light/dark cycle |
|  | 15:00-15:30  Tail suspension | 15:30-17:30 Stroboscopic light | 16:00-17:00 Restraint stress | 15:30 -16:30  Restraint stress | 15:00-17:00  Loud noise |  |  |
|  | 20:00-21:00 Stroboscopic light | 20:30-9:30 Tilted cage | 19:00-21:00  Loud noise | 21:00-9:30  Tilted cage |  |  |  |
| Week8 | 11-13h  Loud noise | 10:30-12:30h Stroboscopic  light | 11-13h  Loud noise | 10:00-12:00  Loud noise | 10:30-12:30 Stroboscopic light | Inversion  light/dark cycle | Inversion  light/dark cycle |
|  | 15:00-17:00 Stroboscopic light | 15:00-17:00  Loud noise | 15:00-17:00 Stroboscopic light | 15:30 -16:30  Restraint stress | 15:00-17:00  Loud noise |  |  |
|  | 20；00-10;00  Tilted cage | 19:30-21:00 Restraint stress | 20；00-10;00  Tilted cage | 21:00-9:30  Tilted cage |  |  |  |

**TABLE S1.** Chronic Mild Stress Protocol

Reference

[1] Branca JJV, Carrino D, Paternostro F, Gulisano M, Becatti M, Di Cesare Mannelli L, Pacini A. Antioxidant support to ameliorate the oxaliplatin-dependent microglial alteration: morphological and molecular study. Eur J Histochem. 2021 Nov 10;65(s1):3285.

[2] Takeshita M, Banno Y, Nakamura M, Otsuka M, Teramachi H, Tsuchiya T, Itoh Y. The pivotal role of intracellular calcium in oxaliplatin-induced inhibition of neurite outgrowth but not cell death in differentiated PC12 cells. Chem Res Toxicol. 2011 Nov 21;24(11):1845-52.

[3] Sun Y, Sukumaran P, Singh BB. Magnesium-Induced Cell Survival Is Dependent on TRPM7 Expression and Function. Mol Neurobiol. 2020 Jan;57(1):528-538.

[4] Zhao Q, Wu X, Yan S, Xie X, Fan Y, Zhang J, Peng C, You Z. The antidepressant-like effects of pioglitazone in a chronic mild stress mouse model are associated with PPARγ-mediated alteration of microglial activation phenotypes. J Neuroinflammation. 2016 Oct 4;13(1):259.
